# 

**Published:** 1843-04-15

**Authors:** 


### CONTENTS.

A Course of Clinical Lectures, delivered at the Hôtel-Dieu, Paris, for the Session 1842–'43. By A. F. Chomel. Lecture 5.
Severe double pneumonia, complicated with enteritis—Recovery. Remarks on some points in the diagnosis of pneumonia, . . . . . . 73
Dr. Tucker's Case of Abscess, . . 74
Chapped Nipples, . . . . . 75

*Jefferson Medical College.*
Clinic of Professor Mütter.
Case. Ranula—Operation, . . 76

*Bibliographical Notices.*
The Annual Report of the Court of Directors of the Western Lunatic Asylum to the Legislature of Virginia, with the Report of the Superintendent and Physician. For 1842, . . . 77
A Treatise on Ruptures. By W. Lawrence, F.R.S., etc. etc. . . . 78
A System of Practical Surgery. By William Fergusson, F.R.S.E., Professor of Surgery in King's College, London, etc. etc. With 246 Illustrations, from drawings by Baggs, engraved by Gilbert. With Notes and Additional Illustrations. By George W. Norris, M. D., Surgeon to the Pennsylvania Hospital, . . . 79

*Editorial.*
Medical Ethics, . . . . 79

*Retrospect of the Medical Sciences.*
On the use of the Liquor of Hydriodate of Arsenic and Mercury in Cutaneous and Uterine Affections, . . . 80
Extirpation of a Tumor involving the Parotid Gland, . . . . . 80
Remarks on Abnormal Sounds in the Ear, . . . . . . . . 81
Fracture of the Femur in a Female 89 years old, . . . . . . 82
A Tabular view of the Treatment of Uterine Hæmorrhages, . . . 83
Cure of Glanders in Man, . . . 84
Accidents in Coal Mines, . . . 84
Hunter's Operation for Aneurism , . 84

